# Food-based interventions to mitigate household food insecurity in Canada: a systematic review

**DOI:** 10.24095/hpcdp.45.9.03

**Published:** 2025-09

**Authors:** Leanne Idzerda, Calin Lazarescu, Tricia Corrin, Eric Vallires, Alix Couture, Sara Khan, Lynn McIntyre, Valerie Tarasuk, Alejandra Jaramillo Garcia

**Affiliations:** 1 Centre for Surveillance and Applied Research, Public Health Agency of Canada, Ottawa, Ontario, Canada; 2 National Microbiology Laboratory, Public Health Agency of Canada, Guelph, Ontario, Canada; 3 Regional Operations Quebec Region, Public Health Agency of Canada, Montral, Quebec, Canada; 4 Environmental Health Science and Research Bureau, Health Canada, Toronto, Ontario, Canada; 5 Cumming School of Medicine, University of Calgary, Calgary, Alberta, Canada; 6 Department of Nutritional Sciences, University of Toronto, Toronto, Ontario, Canada

**Keywords:** household food insecurity, food bank, food charity, food-based intervention, systematic review

## Abstract

**Introduction:**

Household food insecurity (HFI) is a persistent and important public health and policy concern within Canada that continues to be widespread in the face of economic uncertainties and inflation. The objective of this systematic review was to synthesize the evidence on food-based interventions that could reduce HFI in Canada.

**Methods:**

Studies that assessed a food-based intervention that might reduce food insecurity and measured HFI were included, regardless of whether that was the primary purpose of the study. Four databases were searched up to 19 February 2025. Screening of abstracts and full texts, data extraction, assessments of risks of bias and certainty of the evidence were conducted independently by two reviewers. PROSPERO CRD42021254450.

**Results:**

Exposure to food voucher programs may reduce HFI, but exposure to food box, community gardening, school food, hunting and fishing, and food charity programs may have little to no effect on HFI. The rate of utilization of food banks by food-insecure households may be low and depends upon food insecurity level and population group.

**Conclusion:**

Food charities may be a last resort for those in need of short-term access to emergency food (i.e. populations experiencing homelessness). However, given the pervasive nature of HFI as a marker of deprivation, it is unlikely that food-based responses will have a major impact on overall HFI, which is primarily an economic problem. A more comprehensive public policy approach to mitigate HFI is likely required.

**Highlights**

- Participating in food box, community gardening, school food, hunting and fishing and food charity programs may have little to no effect on household food insecurity (HFI) (low certainty).
- Participating in food voucher programs may reduce HFI (low to moderate certainty).
- Food-insecure households’ utilization of food banks is likely low and depends on the severity of food insecurity and the population group (low to moderate certainty).
- The rate and frequency of food bank utilization is likely high among people experiencing homelessness and particularly youth (moderate to high certainty), but these interventions are unlikely to reduce HFI in the long term.
- A comprehensive public policy approach that addresses economic deprivation is likely more effective at reducing HFI (moderate certainty) than food-based interventions.

## Introduction

In 2022, 2.7 million Canadian households experienced food insecurity in the past 12months, including 1.8 million children aged less than 18 years.^1^ This is the highest number recorded in the 17 years since Canada started monitoring household food insecurity (HFI).^1^ These statistics refer specifically to households’ inability to acquire or consume an adequate quantity of food or the uncertainty that they will be able to do so because of financial constraints.^1^

HFI is strongly associated with multiple adverse health outcomes for children and adults in Canada, including heightened nutritional vulnerability;^2^-^7^ increased risk of type 2 diabetes;^8^ poorer mental health;^9^-^15^ higher rates of infectious^16^ and noncommunicable^10^ diseases, injuries^17^ and chronic pain;^18^ poorer disease management;^16^,^19^-^22^ higher rates of health care utilization;^23^-^28^ and premature mortality.^29^,^30^ These relationships are graded, with more severe food insecurity associated with both a higher number of and worse health outcomes^23^-^25^,^27^ even after controlling for income and other sociodemographic characteristics.

It is worth noting that HFI, as it is defined in this manuscript and by public policy in Canada, is not the absence of food security. According to the Food and Agriculture Organization, “food security exists when all people, at all times, have physical and economic access to sufficient, safe and nutritious food to meet their dietary needs and food preferences for an active and healthy life.”^31^^,p.1^ Chronic HFI is a narrower concept and a strong, validated indicator for social policy and population health.^1^ This construct is distinct from broader definitions related to community food security that include constructs of physical, social and economic access to sufficient, safe and nutritious food that meet peoples’ dietary needs and preferences.^32^

Both public policy and food-based interventions have been proposed to reduce HFI. Public policy interventions address the upstream economic determinants of HFI and include indirect supports such as cash transfers, housing assistance and market subsidies. Food-based interventions address shortages of food at the household level. Food-based interventions include food charities that distribute food directly to people, for example, via food banks and soup kitchens, community food programs such as cooking classes, debt counselling for better food budget management, community kitchens and community gardening programs. These programs intend to improve access to food (e.g. by providing free or reduced-cost food), thus reducing the need to expend household resources on food purchases, or they aim to increase program participants’ abilities to manage scarce resources (e.g. by increasing cooking and shopping skills or addressing debt).

There has been little research on the effectiveness of these interventions in reducing HFI in Canada.^33^ A synthesis of the available evidence is particularly important given the surge in federal and provincial government funding of these programs throughout the COVID-19 pandemic^34^ and a parallel increase in the cost of living since 2022.^35^ This policy response is not new, with some food-based interventions such as food banks dating back to the 1980s.^36^

The objective of this systematic review is to synthesize the evidence on the effectiveness of food-based strategies to reduce HFI in Canada.

## Methods

***Systematic review registration***

This systematic review was guided by the *Cochrane Handbook for Systematic Reviews of Interventions*^37^ and followed the Preferred Reporting Items for Systematic Reviews and Meta-Analyses (PRISMA) Guideline.^38^ The initial overarching research question was, “What interventions are effective in reducing HFI in Canada?” 

The systematic review protocol was created a priori and registered in PROSPERO (CRD42021254450).

During the systematic review process and after discussions with HFI experts [VT, LM], it became clear that it would be best to group the interventions into two categories based on the level at which they work, that is, public policy interventions versus food-based interventions. Given that these are distinct intervention categories, this separation facilitated analysis and reporting. Public policy interventions address the underlying determinants of food insecurity and target the economic vulnerability of households. Food-based interventions address food shortages at the household level. The analyses and reporting were conducted separately for the two types of interventions. The systematic review on public policy interventions has been published elsewhere.^39^

This current systematic review summarizes the evidence on food-based interventions to reduce HFI and answers two key questions. The first of these key questions, KQ1, asked, “What is the impact of exposure to a food-based intervention on HFI in Canada?”

Given the low number of studies reporting on the effectiveness of food charity interventions and evidence that food bank utilization rates are low, we included studies of the utilization of food charity interventions by food-insecure households. The second key question, KQ2, reflects the inclusion of these studies: “What is the rate and frequency of utilization of food charity interventions in Canada among food-insecure households?”

***Eligibility criteria***

The population, interventions, comparators and outcomes (PICO) model was used to facilitate search strategy development.

- Population: Households (KQ1) or food-insecure households (KQ2) in Canada.
- Intervention or utilization: Studies that sampled households that were exposed to (KQ1) or that utilized (KQ2) an intervention with the aim of reducing HFI, regardless of whether that was the primary aim of the study.
- Control: Studies with a comparator group (contemporaneous, historical or where participants act as their own control) (KQ1) or no comparator group (KQ2).
- Outcome: Any outcome that aimed to assess HFI or a construct aligned with hunger (KQ1) or the use of food charity interventions (KQ2).

Other eligibility criteria include the following:

- Dates: All studies published from 2000 onwards.
- Languages: English and French.
- Study design: Primary research studies, including controlled trials and observational studies.

For a full list of inclusion and exclusion criteria, refer to Supplementary Material A.

***Search strategy***

The search strategy was developed by a Health Canada research librarian in collaboration with the authors (see Supplementary Material B for details of the search strategy). The search strategy underwent a Peer Review of Electronic Search Strategies,^40^ and was independently reviewed for quality by a second librarian.

We conducted searches in the following four electronic bibliographic databases: EconLit, Embase, Ovid MEDLINE and Scopus. The search was conducted in April 2021 and updated in November 2022, October 2023 and February 2024 and, finally, on 19 February 2025. We conducted a grey literature search using Google Scholar and a targeted website search for key terms in June 2021. In addition, the reference lists of 17 related reviews were hand searched, and experts were consulted to ensure that the database searches did not miss any studies.

***Study selection***

Search results were imported into the web-based literature review software DistillerSR version 2.37 (DistillerSR Inc., Ottawa, ON, Canada), and duplicates were removed. Two reviewers [LI, TC, AC, EV, SK or CL], working independently, screened all titles and abstracts for potential eligibility using a standardized form developed a priori and piloted by all these reviewers. The reviewers then conducted full-text screenings of the retrieved articles that had passed this first stage of screening. (For a list of excluded studies and the reasons for exclusion at either stage of screening, see Supplementary Material C.)

Disagreements between reviewers were resolved through discussion, with a third reviewer if necessary.

The process was similar for decisions regarding data extraction, risk of bias and Grading of Recommendations, Assessment, Development and Evaluation (GRADE).

***Data extraction***

A data extraction form for recording relevant information from each included study was developed and piloted by reviewers prior to starting data extraction. For all included studies, the following information was extracted: citation information (authors, title, journal or source, year of publication, language of publication); study information (objectives, study design, time period, description of intervention and the method or tool used to measure HFI); participant characteristics (including any subgroups of interest); and outcomes of interest (HFI severity and utilization rates of food charities).

***Data analysis***

The final dataset was exported to Microsoft Excel 365 (Microsoft Corp., Redmond, WA, US) for analysis. Narrative synthesis was performed on the dataset. Where three or more studies measured the same outcome, a random-effects meta-analysis using the DerSimonian and Laird method^37^ weighting procedure was conducted in the metaprop package in Stata version 18 (StataCorp LLC, College Station, TX, US). Logit transformation was used and the data were then subgrouped by population and level of HFI. Heterogeneity was only calculated when there were four or more observations. Subanalyses by sociodemographic factors were included where possible.

***Risk of bias***

We used the Risk of Bias in Non-randomised Studies – of Intervention (ROBINS-I)^41^ and Risk of Bias in Non-randomized Studies – of Exposures (ROBINS-E)^42^ tools and the Cochrane Collaboration’s tool for assessing risk of bias in randomized trials version 2 (RoB 2)^43^ to assess risk of bias in the included studies that examined the effectiveness of food-based interventions (KQ1). The JBI critical appraisal tool for cross-sectional studies was used to assess risk of bias in studies of food charity utilization (KQ2) as cross-sectional data were extracted from all of the included studies.^44^,^45^

***Certainty of the evidence***

The GRADE framework was used to rate the certainty and strength of the body of evidence.^46^ The purpose of GRADE is to rate the quality of the evidence in relation to research questions in a systematic and transparent manner (see Supplementary Material D for the GRADE decision rules applied in this systematic review). The evidence in the studies that addressed KQ1 was first assumed as being of high certainty, and this was rated down as per recent guidance on non-randomized studies.^47^ The studies that addressed KQ2 were also initially assumed to have high-certainty evidence as an adapted GRADE approach was used to assess them.^48^ In assessments of rates and frequency of utilization, observational studies can provide robust estimates when they use appropriate measures and enrol representative populations.

The certainty was downgraded for each outcome to “moderate,” “low” or “very low” if there were serious or very serious concerns that reduced certainty in the outcome estimates across the following five domains: risk of bias, inconsistency, indirectness, imprecision or publication bias.^47^ The wording of the summary statements regarding the certainty of the evidence is based on published guidance.^49^

## Results

**Figure 1:**
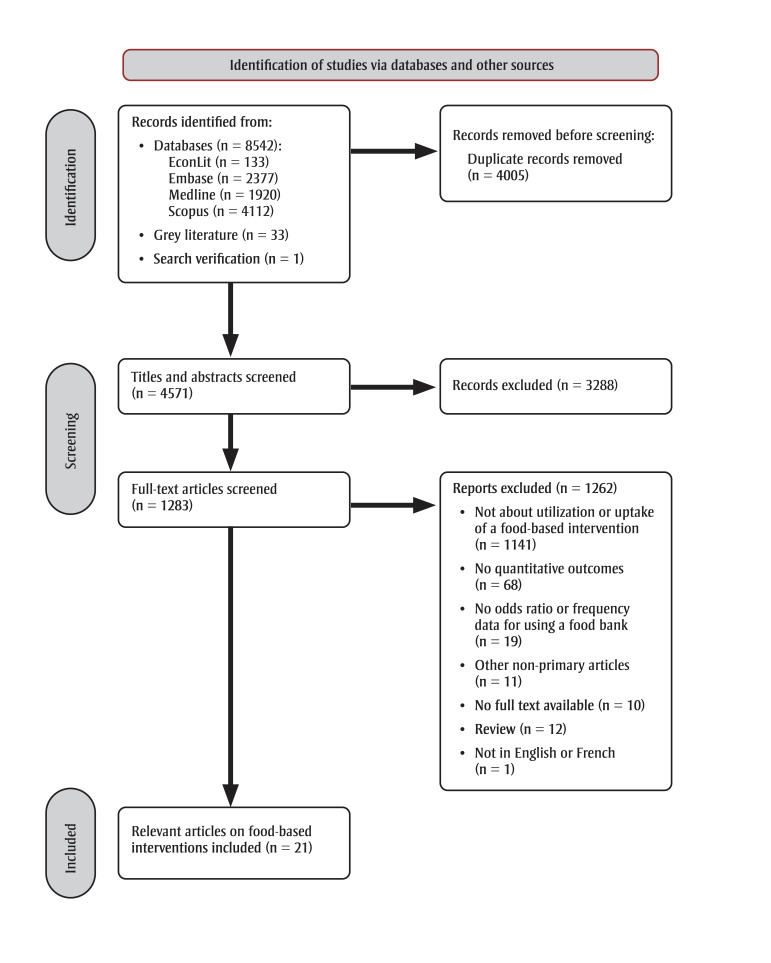
PRISMA flow diagram of searches of databases and other sources

**Abbreviation: **PRISMA, Preferred Reporting Items for Systematic Reviews and Meta-Analyses. 

A total of 8542 references were screened for eligibility for both key questions. Of these, 21 articles reported on exposure to or utilization of food-based interventions intended to reduce HFI in Canada (Figure 1).

***Descriptive summary of included studies***

The included studies (n=21) were published between 2000 and 2023, with more than half (n=13) published since 2014 (see Table 1 for a summary of the study characteristics). Ten studies reported on the impact of exposure to food-based interventions on HFI (KQ1), and 13 on the rates and frequency of utilization of food-based interventions by food-insecure households (KQ2); 2 of these studies reported on both the impact and utilization rate of food-based interventions. The characteristics of the studies are summarized in Table 1. Detailed risk of bias results for all studies are in Supplementary Material E.

**Table 1:** Characteristics of all included studies, by program type (n = 21)

First author, publication year Study design Dataset	Location Period of data collection Intervention	Population	Comparator	Food insecurity collection method Reference time period	Total sample size, n	Risk of bias
Food voucher programs						
Aktary, 2024^50^ Randomized controlled trial Data collected by the authors for the purpose of this study	British Columbia 2019 Farmers’ Market Nutrition Coupon Program	Adults living with low income who have never participated in the Farmers’ Market Nutrition Coupon Program	Adults living with low income who do not receive the Farmers’ Market Nutrition Coupon Program coupons	HFSSM Past 30 days	285	Low
Heasley, 2021^51^ Single-arm pre–post design Data collected by the authors for the purpose of this study	Guelph, Ontario 2019–2020 Food prescription voucher program	Patients with at least 1 diagnosed cardiometabolic condition or micronutrient deficiency (or both) and experiencing food insecurity	Participants pre-intervention	HFSSM Past 4 months	60	High
Food box programs						
Miewald, 2012^52^ Cohort (2-arm pre–post design) Data collected by the authors for this study	British Columbia Baseline: 2008; followup: 8 months after baseline Food subscription box	Households with low incomes that received a weekly food box	Households with low incomes that accessed the same social services but did not receive a weekly food box	HFSSM Not specified^a^	192	High
Gardening programs						
Sandha, 2021^53^ Analytic cross-sectional Data collected by the authors for the purpose of this study	Prince Edward Island 2013 At-home gardening	Mothers of children aged 0–6 years who accessed the Family Resource Centre’s gardening program	Mothers of children aged 0–6 years who did not access the Family Resource Centre’s gardening program	HFSSM Reference time period not specified^a^	282	Some concerns
School food programs						
Roustit, 2010^54^ Analytic cross-sectional Health and Social Survey of Qubec Children and Youth	Quebec 1999 School food programs (free or reduced-price snacks or meals)	Children and adolescents attending a primary or secondary school with a food supplementation program	Children and adolescents attending a primary or secondary school without a food supplementation program	Three statements from the Radimer/Cornell questionnaire Reference time period not specified	2346	High
Hunting and fishing interventions specific to Indigenous communities						
Blanchet, 2021^55^ Analytic cross-sectional Data collected by the authors for the purpose of this study	Syilx People of the Okanagan Nation, British Columbia 2018 Salmon consumption	Adults who self-identified as or were in a kin relationship with a person who self-identified as Syilx of the Okanagan Nation and who eat locally caught salmon	Adults who self-identified as or were in a kin relationship with a person who self-identified as Syilx of the Okanagan Nation and who eat non-locally caught salmon or no salmon	HFSSM adapted to Indigenous populations in Canada Not specified^a^	265	High
Thompson, 2012^56^ Analytic cross-sectional Data collected by the authors for the purpose of this study	Manitoba 2008–2012 Country Food Programs	Adults in 14 remote communities in northern Manitoba	NA	HFSSM and 3 supplemental questions on gardening, hunting and fishing Not specified^a^	533	High
Food charity interventions						
Loopstra, 2012^57^^b^ Longitudinal cohort (single arm) Data collected by the authors for the purpose of this study	Toronto, Ontario Baseline: 2005–2007; followup: 2006–2008 Local food banks	Adults in families with low incomes, based on census tracts, who used a food bank in the last 12 months	Adults in families with low incomes, based on census tracts, who did not use a food bank in the last 12 months	HFSSM Past 12 months	371	High
Rizvi, 2021^58^^b^ Longitudinal cohort (single arm) Data collected by the authors for the purpose of this study	Ottawa, Ontario 2017–2019 Local food banks	People who accessed community food banks, after 6, 12 and 18 months	People who accessed community food banks, at baseline	HFSSM Past 12 months	401	High
Roncarolo, 2016^59^ Longitudinal cohort (2 arms) Data collected by the authors for the purpose of this study	Montral, Quebec Baseline: 2011–2012; followup: 9 months after baseline Local food banks	Individuals accessing traditional food banks or participating in alternative community interventions (community kitchens, community gardens and buying groups)	Population prior to food bank interventions	HFSSM Past 12 months	824	High
Food charity utilization						
Daly, 2023^60^ Cross-sectional Assessing the Impacts of COVID-19 on Mental Health survey	National 2020–2021 Local food charities	Adults in food-insecure households stressed or worried about having enough food to meet the household’s basic needs as a result of the COVID-19 pandemic in the past 2 weeks	NA	One question on food worry and one question from the HFSSM Past 12 months	477	Low
Holmes, 2019^61^ Cross-sectional Data collected by the authors for the purpose of this study	Vancouver, British Columbia 2015 Local food banks	Food-insecure households that accessed a local food bank	NA	HFSSM Not specified^a^	77	High
Kirkpatrick, 2009^62^ Cross-sectional Data collected by the authors for the purpose of this study	Toronto, Ontario 2005–2007 Local food banks	Food-insecure households	NA	HFSSM Past 12 months	484	High
Loopstra, 2012^57^^b^ Cross-sectional data taken from a cohort study Data collected by the authors for the purpose of this study	Toronto, Ontario Baseline: 2005–2007; followup: 2006–2008 Local food banks	Adults in families with low incomes, based on census tracts, who used a food bank at least once in the last 12 months	Adults in families with low incomes, based on census tracts, who did not use a food bank in the last 12 months	HFSSM Past 12 months	371	Low
MacBain, 2023^63^ Cross-sectional Data collected by the authors for the purpose of this study	Hamilton, Ontario 2021 Local food charities	Food-insecure households	NA	Hunger Vital Sign tool Past 12 months	173	Low
McIntyre, 2000^64^ Cross-sectional NLSCY Cycle 1	National (excluding the territories) 1994 Local food banks	Households with children aged less than 18 years	NA	Single question on child hunger Any experience (“ever experienced being hungry”)	16 639	Some concerns
McIntyre, 2012^65^ Repeated cross-sectional NLSCY Cycles 2 and 7	National (excluding the territories) 1996–1997; 2006–2007 Local food banks	Households with children aged 2 to 9 years	NA	Single question on child hunger Any experience (“ever experienced being hungry”)	Cycle 2: 8165 Cycle 7: 15 691	Some concerns
Men, 2021^66^ Cross-sectional Canadian Perspectives Survey Series (CPSS)	National (excluding the territories) 2020 Any charitable food intervention	Canadians living in the 10 provinces	NA	Six-item questionnaire adapted from the HFSSM Past 30 days	4410	Low
Parpouchi, 2016^67^ Cross-sectional Data collected by the authors for the purpose of this study	Vancouver, British Columbia 2009–2011 Any charitable food intervention	Adults with a mental illness who were experiencing homelessness	NA	HFSSM adapted to populations experiencing homelessness Past 30 days	497	Low
Rizvi, 2021^58^^b^ Longitudinal design (cohort) Data collected by the authors for the purpose of this study	Ottawa, Ontario 2017–2019 Local food banks	People who accessed community food banks, after 6, 12 and 18 months	People who accessed community food banks, at baseline	HFSSM Past 12 months	401	Low
Tarasuk, 2009^68^ Cross-sectional Data collected by the authors for the purpose of this study	Toronto, Ontario 2003 Any charitable food intervention	Youth aged 16–24 years without stable or secure housing	NA	Modified version of the HFSSM Past 30 days	261	Low
Tarasuk, 2020^69^ Cross-sectional Canadian Household Panel Survey (CHPS) pilot	Saskatchewan, Ontario, Quebec and New Brunswick 2008 Local food banks	Households (except those on reserves, in religious and other communal colonies or in institutions, and members of the Canadian Forces)	NA	One question from the HFSSM Past 12 months	1593	Low
Vahabi, 2011^70^ Cross-sectional Data collected by the authors for the purpose of this study	Toronto, Ontario 2008 Local food banks	Spanish- or Portuguese-speaking adults aged 20 years or older who had immigrated to Canada in the past 5 years from Central or South America	NA	One question from the HFSSM (translated into Spanish and Portuguese) that asked if participants ever had to eat less because they did not have enough money to buy food Past 12 months	70	Low

**Abbreviations:** HFSSM, Household Food Security Survey Module; NLSCY, National Longitudinal Survey of Children and Youth; NA, not applicable.

^a^ Although not specified, it was assumed that the HFSSM time period was 12 months because no adaption of the module was reported. 

^b^ Included in both the food charity intervention and food charity utilization sections of this table as reported data are relevant to both. 

***The impact of exposure to food-based interventions on HFI in Canada (KQ1)***

Food-based interventions included food charity programs (n= 3),^57^-^59^ food voucher programs (n=2),^50^,^51^ hunting and fishing programs specific to Indigenous communities (n=2),^55^,^56^ food box programs (n=1),^52^ gardening programs (n=1)^53^ and school food programs (n=1).^54^ Overall, low-certainty evidence suggests that participation in food box, gardening, hunting and fishing, school food and food charity programs may have little to no effect on HFI. Low- to moderate-certainty evidence suggests that food voucher programs may reduce food insecurity. The findings are summarized in Table 2 and described in detail in the following subsections.

**Table 2:** Summary of findings of studies examining the impact of exposure to food-based interventions on HFI in Canada, by program type (KQ1)

First author, publication year Study design (number of studies)	Population Period of data collection	Outcome (food insecurity level)	Number of participants exposed to intervention, n	Number of participants not exposed to intervention, n	Effect size	Direction of effect	Certainty of evidence
Food voucher programs							
Aktary, 2024^50^ Randomized controlled trial	Adult population living with low income in British Columbia 2019	Total HFI (marginal, moderate and severe)	143	142	Post intervention: OR = 0.21 (0.06–0.70); 16-week followup: OR = 0.29 (0.09–0.96)	Favours intervention (participation in Farmers’ Market Nutrition Coupon Program)	Moderate^a^^b^
Heasley, 2021^51^ Single-arm pre–post design	Food-insecure households in Guelph, Ontario 2019–2020	Total HFI (marginal, moderate and severe)	60	NA	OR = 0.18 (0.07–0.50)	Favours intervention (participation in food prescription voucher program)	Low^a^^b^^c^
Food box programs							
Miewald, 2012^52^ Cohort (2-arm pre–post design)	Households with low incomes in British Columbia 2008	Moderate and severe	46	44	No effect	NA	Low^a^^b^^c^
Gardening programs							
Sandha, 2021^53^ Analytic cross-sectional	General population in Prince Edward Island 2013	Total HFI (marginal, moderate and severe)	104	175	No effect	NA	Low^a^^b^^c^
School food programs							
Roustit, 2010^54^ Analytic cross-sectional	Primary or secondary school-aged children and adolescents in the general population in Quebec 1999	Moderate and severe	678	1524	No effect	NA	Low^a^^c^
Hunting and fishing interventions specific to Indigenous communities^d^							
Blanchet, 2021^55^ Analytic cross-sectional	Syilx of the Okanagan Nation adults 2018	Total HFI (marginal, moderate and severe)	612	88	No effect	NA	Low^a^^c^^e^
Thompson, 2012^56^ Analytic cross-sectional	Adults in remote communities in northern Manitoba 2008–2012	Moderate and severe					
Food charity interventions^f^							
Loopstra, 2012^57^ Cohort (2-arm pre–post design) 2005–2007; 2006–2008	Families living with low income in Toronto, Ontario	Moderate and severe	85	286	No effect	NA	Low^a^^c^^e^
Roncarolo, 2016^59^ Longitudinal cohort (2 arms) 2011–2012	People who accessed food banks in Montral, Quebec	Moderate and severe	372 (traditional food banks)	78 (alternative interventions, i.e. community kitchens, community gardens, buying groups)	OR = 0.44 (0.29–0.67) (for traditional food banks) No effect (for alternative food programs)	Favours intervention (traditional food bank)	
Rizvi, 2021^58^^g^ Longitudinal cohort (single arm) 2017–2019	People who accessed community food banks in Ottawa, Ontario	Moderate and severe	401	NA	No effect	NA	

**Abbreviations: **GRADE, Grading of Recommendations, Assessment, Development and Evaluation; HFI, household food insecurity; NA, not applicable; OR, odds ratio.

^a^ Indirectness: Study population not indicative of general population. 

^b^ Imprecision: Optimal information size not met. 

^c^ Risk of bias: Study had either high risk of bias or some concerns related to risk of bias.

^d^ The population, intervention, outcomes, and study design in these studies were deemed similar enough that they could be combined.

^e^ Inconsistency: Inconsistent effect estimates and direction of effect.

^f^ The certainty of evidence of these studies was assessed as a single group as the populations and interventions are similar enough for the GRADE ratings. The data were not combined due to the difference in study designs.

^g^ Rizvi et al.58 conducted a longitudinal study but also assessed aggregated data from all time points.

**Food voucher programs**

In a randomized controlled trial, adults in households with low incomes received coupons valued at CAD21 each week for 10 to 15 weeks to purchase healthy foods from local farmers’ markets in British Columbia.^50^ Aktary et al. reported that the odds of experiencing short-term HFI were 79% lower at the end of intervention (*p*=0.01) and 71% lower at 16 weeks postintervention (*p*=0.04) in the group receiving the coupon compared to the control group.^50^

In a pre- and postintervention single-arm study conducted in Guelph, Ontario, community health centre patients with a diagnosed cardiometabolic condition, a micronutrient deficiency or both and who were experiencing food insecurity were prescribed 12 weekly vouchers to use at community food markets.^51^ Heasley et al. reported that the mean adult and child food insecurity scores decreased significantly from baseline to followup (*p* <0.001 for adults; *p*=0.01 for children).^51^

**Food box programs**

In a cohort study conducted in Vancouver, British Columbia, Miewald et al. assessed the effects of monthly distributions of food boxes of fruits and vegetables over an 8-month period to people in households with low incomes or in areas with poor food access or to older adults assessed.^52^ There was no statistically significant change in HFI among those who enrolled in the food box program versus those who did not.^52^

**Hunting and fishing interventions specific to Indigenous communities**

Two analytic cross-sectional studies assessed interventions specific to Indigenous communities. Blanchet et al. found no association between the type of salmon eaten (locally caught salmon versus imported or no salmon) and HFI among Syilx of the Okanagan Nation adults in British Columbia.^55^

Thompson et al. assessed households’ access to hunted and fished foods in 14 remote Indigenous communities in northern Manitoba and found no association between access to these foods and HFI.^56^ In the same study, the presence of a country food program (OR=20.6; 95%CI: 2.4–176.1), road access to urban areas (OR=7.6; 95% CI: 1.2–51.5) and access to a public transit system (OR=3.9; 95%CI: 1.5–9.9) were all associated with lower rates of HFI, whereas living in a geographically compact community was not associated with lower rates of HFI.^56^

**Gardening programs**

In an analytic cross-sectional study conducted in Prince Edward Island, Sandha et al. compared the levels of HFI of mothers with children aged 0 to 6 years who accessed Family Resource Centre gardening programs with those of mothers with children the same age who did not access these programs. The authors reported that there was no relationship between HFI and access to the gardening progam.^53^

**School food programs**

In a cross-sectional study, Roustit et al. assessed elementary and secondary school food supplementation programs in Quebec and found no relationship between exposure to these programs and HFI.^54^ Students in schools without (10.4%) and with (12.7%) these supplementation programs were food insecure (*p*=0.22).^54^

**Food charity interventions**

Three studies assessing four food charity interventions were included.^57^-^59^ The interventions included both traditional food bank models (where food bank clients receive a hamper of food regularly, usually once per month) and alternative community-based food charities (where participants can, for example, “shop” for groceries at a food bank or take part in community kitchen programs).

In a study that evaluated changes to HFI over a 9-month period, Roncarolo et al. found that severe HFI significantly decreased among participants in Montral, Quebec, who accessed a traditional food bank (from 89.6% to 61.1%; OR = 0.27; 95% CI = 0.14–0.54 for severely food-insecure households), but did not significantly decrease among those who participated in alternative interventions (community kitchens, community gardens and buying groups).^59^

In a longitudinal study, Rizvi et al. found that most of the people accessing food banks in Ottawa, Ontario, were food insecure at baseline and remained food insecure at the 18-month followup, although there was a small downward trend in the proportion of people who were severely food insecure (from 38.5% to 24.6%; no measure of significance reported).^58^

In a study of families with low incomes accessing food banks in Toronto, Ontario, Loopstra and Tarasuk reported that 13.0% were no longer severely food insecure, 40.7% remained severely food insecure and 9.3% had become severely food insecure at followup 1 year post-baseline, for a net change of 3.7%.^57^

***Rates of food charity utilization among food-insecure households (KQ2)***

Eleven studies reported on rate (n=11)^57^,^60^,^62^-^70^ of utilization of food charity interventions among food-insecure households.

**Figure 2:**
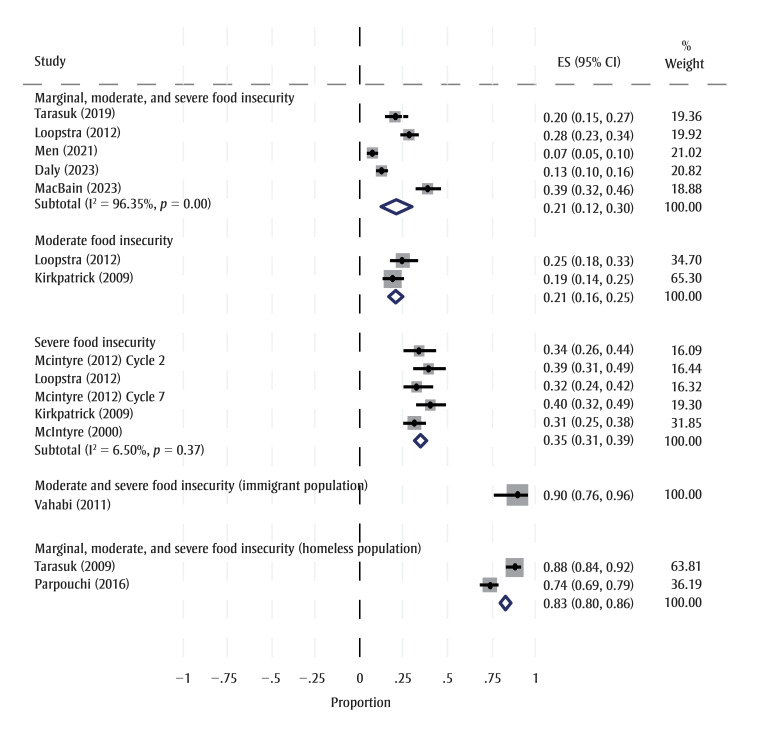
Forest plot for rates of food charity utilization by HFI level, Canada (KQ2)

**Abbreviations:** CI, confidence interval; ES, effect size; HFI, household food insecurity; I2, measure of heterogeneity. 

The rate of food-insecure households that used a food charity varied by severity of HFI and population group (Table 3; Figure 2). Those with severe HFI generally accessed food charities more often than those with moderate levels of HFI (Figure 2). The rate of food charity utilization was highest among those who were precariously housed or experiencing homelessness (Table 3; Supplementary Material F).

**Table 3:** Summary of findings of the rate of food charity utilization by food-insecure households according to HFI level, Canada (KQ2) (n = 11)

First author, publication year Study design	Period of data collection	Sample size who used a food charity, n	Sample size who did not use a food charity, n	Meta-analysis subtotal, %^a^	Interpretation of effect	GRADE
Marginal, moderate and severely food insecurity						
Tarasuk, 2020^69^ Cross-sectional	2008	276	1342	21	A small proportion of households that were food insecure used a food charity	Moderate^b^^c^
Loopstra, 2012^57^ Cross-sectional	2005–2007, 2006–2008					
Men, 2021^66^ Cross-sectional	2020					
Daly, 2023^60^ Cross-sectional	2020–2021					
MacBain, 2023^63^ Cross-sectional	2021					
Moderately food-insecure households						
Loopstra, 2012^57^ Cross-sectional	2005–2007, 2006–2008	63	237	21	A small proportion of households that were food insecure used a food charity	Low^c^^d^^e^
Kirkpatrick, 2009^62^ Cross-sectional	2005–2007					
Severely food-insecure households and child hunger						
McIntyre, 20126^65^ Repeated cross-sectional	1996–1997, 2006–2007	230	427	35	A small proportion of households that were food insecure used a food charity	Moderate^c^^d^
Loopstra, 2012^57^ Cross-sectional	2005–2007, 2006–2008					
Kirkpatrick, 2009^62^ Cross-sectional	2005–2007					
McIntyre, 2000^64^ Cross-sectional	1994					
Moderately and severely food-insecure household within immigrant populations						
Vahabi, 2011^70^ Cross-sectional	2008	34	5	90	A high proportion of this immigrant population used a food charity	Low^b^^c^^e^
Marginally, moderately and severely food-insecure populations experiencing homelessness						
Parpouchi, 2016^67^ Cross-sectional	2009–2011	431	101	83	A very high proportion of people experiencing homelessness used a food charity	High^c^
Tarasuk, 2009^68^ Cross-sectional	2003					

**Abbreviations:** GRADE, Grading of Recommendations, Assessment, Development and Evaluation; HFI, household food insecurity. 

^a^ Represents the combined data of the meta-analysis (see Figure 2).

^b^ Inconsistency: Inconsistent effect estimates and direction of effect. 

^c^ Indirectness: Study population not indicative of general population.

^d^ Risk of bias: Study had either high or moderate/some concerns risk of bias.

^e^ Imprecision: Optimal information size not met.

***Frequency of food charity utilization by food-insecure households (KQ2)***

Six studies^57^,^58^,^60^,^62^,^66^,^68^ assessed the frequency of utilization of food charity interventions by food-insecure individuals. Although there was no consistent measurement of the frequency of utilization of food charities or food banks, it was clear that the frequency of food bank use among food-insecure households was low (Table 4). Youth aged 16 to 24 years experiencing homelessness appear to use charitable meal programs (soup kitchens, outreach vans, drop-in centres, shelters) more frequently than households that are food insecure.

**Table 4:** Summary of findings of frequency of food charity utilization, Canada (KQ2) (n = 6)

First author, publication year Study design Population	Food insecurity levels	Size of food-insecure population, n	Percentage of the food-insecure population, %	Frequency	GRADE
Holmes, 2019^61^ Cross-sectional Food-insecure households	Severe	43	70	Low (< 1 visits/month)	Low^b^^c^^d^
			68	Medium (1–2 visits/month)	
			64	High (> 2 visits/month)	
Kirkpatrick, 2009^62^ Cross-sectional Food-insecure households	Moderate	182	18.7	Used a food bank at least once in the previous 12 months	
	Severe	134	40.3		
	Moderate	182	5	Used a food bank at least once in 10 or more of the previous 12 months	
	Severe	134	6.7		
Loopstra, 2012^57^ Cross-sectional Food-insecure households	Marginal	47	10	Used a food bank at least once in the previous 12 months	
	Moderate	118	27		
	Severe	112	39		
Men, 2021^66^ Cross-sectional Food-insecure households	Total HFI (marginal + moderate + severe)	540	7.4	Received charitable food assistance at least once in the past 30 days	
			4.3	Received charitable food assistance more than once in the past 30 days	
Rizvi, 2021^58^ Cross-sectional^a^ Food-insecure households	Marginal	226	24.3	Used a food bank once in the last 3 months	
	Moderate	444	22.6		
	Severe	408	22.0		
	Marginal	226	22.6	Used a food bank twice in the last 3 months	
	Moderate	444	17.7		
	Severe	408	50.4		
	Marginal	226	45.6	Used a food bank 3 or more times in the last 3 months	
	Moderate	444	50.4		
	Severe	408	47.2		
Tarasuk, 2009^68^ Cross-sectional Female youth experiencing homelessness	Moderate and severe	112	27	Used a charitable meal program 1–2 days in the last 7 days	Moderate^e^
			38	Used a charitable meal program 3–5 days in the last 7 days	
			21	Used a charitable meal program 6–7 days in the last 7 days	
Tarasuk, 2009^68^ Cross-sectional Male youth experiencing homelessness		149	22	Used a charitable meal program 1–2 days in the last 7 days	
			57	Used a charitable meal program 3–5 days in the last 7 days	
			11	Used a charitable meal program 6–7 days in the last 7 days	

**Abbreviations: **GRADE, Grading of Recommendations, Assessment, Development and Evaluation; HFI, household food insecurity.

^a^ Rizvi et al.^58^ conducted a longitudinal study but also assessed aggregated data from all time points.

^b^ Risk of bias: Study had either high or moderate/some concerns risk of bias.

^c^ Inconsistency: Inconsistent effect estimates and direction of effect.

^d^ Indirectness: Study population not indicative of general population.

^e^ Imprecision: Optimal information size not met. 

## Discussion

The objective of this systematic review was to synthesize the evidence on food-based interventions to mitigate HFI, recognizing the importance of this indicator for social policy and population health. This systematic review found limited evidence (of low certainty) for interventions assessing the effectiveness of food-based strategies on HFI, except for food voucher programs, for which there was some moderate-certainty evidence favouring intervention. Of note, no evidence was found for some strategies, for example, breakfast clubs or Meals on Wheels programs. Each category of intervention is discussed separately in the following subsections.

***Food charity interventions***

Low-certainty evidence suggests that food charity interventions may result in little to no difference in HFI. One possible explanation is that HFI is not a measure of access to food, but of absolute economic deprivation. A household that cannot afford to buy food likely cannot afford to buy necessary medications, pay the rent or pay for electricity, water and other essentials. Given that food is a basic human need, HFI is a clear marker of a household’s economic resources. Thus, HFI is not about an inability to access food, but rather a measure of economic insecurity. Receiving food charity may have a minimal effect on these economic resources or circumstances. This highlights the need for a more comprehensive economic response to the issue of HFI.

Another possible explanation for this lack of change in HFI is that food charities are limited in the amount of assistance they can provide to any one household. Food charities are neither a consistent nor a reliable coping strategy over the long term and are unlikely to change levels of HFI because households require food every day. Sustaining this level of access to food banks is likely not an economically viable option^71^ as food charities lack the resources to meet clients’ food needs indefinitely.

This systematic review revealed that less than 40% of severely food-insecure households use food banks, and that most of the households that use them access them very infrequently. The low utilization rate may reflect food-insecure households’ recognition that food charities can only provide them with limited assistance. This may be due to inconvenient opening hours or because of a lack of the quantity, quality or type of food they need or prefer, among other reasons. This systematic review also found that increased severity of HFI was associated with higher utilization of food banks. This is in line with previous research that showed that the lack of utilization of these programs likely results from intrahousehold dynamics (such as household economics or coping strategies such as borrowing food and money from friends and family) that cause people to only access food charities when they reach the end of their capabilities.^57^,^65^

Recognition of the stigma associated with using food banks, among other reasons, has resulted in the development of alternative food-charity interventions,^59^ but evidence that these programs reduce HFI is lacking. The findings of this systematic review also bring into question the role of food banks specifically and food charities more broadly in addressing food insecurity. While beyond the scope of this review, the persistence of food charities as a dominant response to HFI would ideally be researched by experts in the field of social science.

We found the proportion of people utilizing food charity programs to be much higher in populations experiencing homelessness, which may highlight the lack of other coping strategies available to them. It is important to note that even with high rates of utilization of food charities, this population continues to face severe and chronic food insecurity. An HFI-reduction strategy that is more comprehensive than solely focusing on provision of food is required.^67^ This notion is corroborated by a recent systematic review that outlined the experience of HFI among people experiencing homelessness in high-income countries.^72^ Easton et al. found that people experiencing homelessness are in a system that maintains food insecurity through oppression (i.e. structural inequities and institutionalism in finding adequate housing), an inability to fulfill basic needs, a lack of facilities for meal preparation and barriers to food assistance such as not having an address or means of identification.^72^ Looking beyond food-based interventions is important to resolve the larger issue of the extreme deprivation of populations experiencing homelessness.

The main reasons for the low certainty of the evidence of the effect of food charities on HFI are the limitations in the study designs (inability to control for concurrent interventions outside the parameters of the study), the small study sample sizes and the significant dropout rates. Large longitudinal prospective cohort studies, such as the Pathways study in Quebec,^73^ will help identify the long-term effects of food charity programs on food insecurity. Without a stronger evidence base for these types of interventions, their effect on HFI in Canada remains unconfirmed. The Office of the Auditor General (OAG) of Canada reached a similar conclusion in their 2021 review of Canada’s pandemic-related expenditures of the food assistance programs intended to mitigate HFI.^74^ The review concluded that lack of data and performance measurement meant that the departments and agencies the OAG audited did not know whether the initiatives had achieved all their outcomes for reducing HFI.^74^ While a lack of evidence does not indicate program ineffectiveness, a growing body of evidence shows that implementing more holistic public policy interventions that address income supports and root economics is effective in reducing HFI.^39^ The evidence thus points towards a more holistic public policy approach that tackles the root economics of the problem in Canada.

***Food box programs***

The evidence in this systematic review suggests that food box programs that regularly supply fresh foods may result in no effect on HFI. The long-term impacts of such programs on HFI may be limited to the lifetime of the interventions, which require sustained and considerable economic input to regularly supply program participants with sufficient fresh food.^71^ This is likely because these interventions, as with all the food-based interventions assessed in this systematic review, do not address the underlying causes of HFI as a marker of economic deprivation.

***Food voucher programs***

This systematic review found that food voucher programs may decrease food insecurity in households with low incomes for the duration of the intervention. This result aligns with those of two other systematic reviews that assessed interventions intended to reduce HFI in Canada and the United States.^75^,^76^ These systematic reviews found that food vouchers were associated with a statistically significant decrease in HFI in both general and clinical populations.^75^,^76^ Supplying households with vouchers may be a way to provide households with the economic resources to purchase culturally appropriate foods and thus increase their purchasing power.

Given the low number of studies that have assessed food voucher programs, more studies are needed to assess their effectiveness as well as the dose–response relationship between duration, frequency of exposure, long-term sustainability and dollar values of these interventions and food insecurity status.

***Interventions specific to Indigenous communities***

This systematic review found low-certainty evidence for the effect of hunting and fishing programs on HFI in Indigenous communities. Food insecurity in Indigenous populations ranges from 48% among on-reserve First Nations communities to 57% among Inuit in Nunavut.^77^ Given these disproportionately high rates of HFI, it is critical to find culturally sensitive and workable solutions that valorize Indigenous ways of knowing. Previous reviews have explored programs and policy interventions related to Indigenous populations,^39^,^78^ while Drysdale et al. synthesized the interventions intended to reduce HFI in remote regions in Canada as well as Australia and the United States.^79^

***Gardening programs***

Although many studies have assessed the potential benefits of gardening interventions, very few measured the effect of these programs on HFI using a validated scale. In a 2022 systematic review that assessed the impacts of community gardens on health in Canada, Japan, the Netherlands, South Africa, the United Kingdom and the United States, Hume et al. concluded that although HFI was not a directly measured outcome, community gardens likely do not affect HFI.^80^ Future studies in this area should include a validated measure of HFI to assess the dimension of community garden and urban agriculture utilization.

***School food programs***

Although school food programs are often touted as an important mechanism for reducing hunger and HFI among youth,^81^ the evidence for their effect on HFI is sparse as most studies on school food programs retrieved for this systematic review were screened out because they did not report on HFI. Two recent reviews by the Alberta Health Services and by Nova Scotia Health independently found that school food programs do not alleviate HFI.^82^,^83^ The Nova Scotia public health authority concluded that school food programs “are not an appropriate or sustainable solution to HFI as they do not address its root causes—primarily, inadequate income.”^83^^,p.5^ These programs may, however, be useful in achieving other outcomes such as improved dietary behaviour and critical food literacy skills (learning, culture and social norms).^84^

***Limitations of the included studies and recommended future research***

**Limited data**

Very few studies of the studies screened used a validated scale to assess the impact of a food-based intervention on HFI or related constructs of hunger, even though the stated purpose was to reduce HFI. Future studies as well as government programming that aims to increase the effectiveness of food-based interventions should include a measure of HFI in their evaluations.

**Intervention variation**

The design and administration of interventions varied considerably, making it difficult to measure their impact on HFI. Studies that assess the effectiveness of different implementations of similar interventions would be beneficial. Authors who report on food charity studies should carefully detail the intervention, the implementation of the interventions and any possible variations. The limited number of studies precluded examination of interjurisdictional variation. However, as there are increasingly large interprovincial differences in food-insecurity prevalence and severity,1 it might be important for future studies to take into account the policy context of these food-based interventions.

**Type of included studies**

A major limitation of this systematic review was the high risk of bias of these largely observational studies and the difficulty in implementing an experimental trial to assess the efficacy of food-based interventions on HFI. The high risk of bias was due to the presence of confounding factors (5 of 9 studies) and to the high levels of missing data because of high dropout rates and loss to followup (4 of 9studies). Assessing the effectiveness of these interventions (assessed through actual utilization rates) also remains a challenge.

**Potential missing studies**

This systematic review only synthesized the evidence that is publicly available and may have missed evidence on other types of food-based interventions that have not been published (e.g. breakfast clubs). In addition, it is also important to report null or negative studies in the literature, to give a fuller picture of the situation.

## Conclusion

Food-based interventions date back more than 40 years,^85^ yet we only retrieved 10 studies that assessed the effectiveness of these interventions on HFI or a hunger-related construct. When categorizing food-based interventions (i.e. school food programs, food charities), we found very few or in some cases no studies of program models that are particularly prominent in Canada, such as children’s breakfast clubs or Meals on Wheels.

The certainty in the evidence that food-based interventions have an effect on HFI is low, indicating that these interventions may not in fact affect HFI. Given the pervasiveness of HFI, and the fact that it is a marker of economic deprivation, it is unlikely that a food-based response will have much of an impact on overall HFI, which is primarily an economic problem. Emerging evidence suggests that more comprehensive public policy approaches are required to mitigate HFI.^39^

## Acknowledgements

The authors wish to thank Bernard Choi, Janet Potvin and Genevieve Garipy, of the Public Health Agency of Canada at the time of their involvement, for helping with the screening process; Mallory Drysdale and Prinon Rahman for helping to assess the risk of bias; and Kate Morissette for helping to undertake the GRADE process. We would like to thank the Health Canada Library for their assistance in designing and implementing the search strategy and the Public Health Agency of Canada Library for the updated search.

## Funding

None. This research received no grants from any funding agency or from the commercial or not-for-profit sectors.

## Conflicts of interest

The authors have no conflicts of interest to declare.

## Authors’ contributions and statement

LI: Conceptualization, investigation, formal analysis, methodology, project administration, supervision, validation, writing— original draft, writing—review and editing.

CL: Formal analysis, investigation, data extraction, project administration, software, writing—original draft, writing—review and editing.

TC: Formal analysis, investigation, writing—original draft, writing—review and editing.

EV: Investigation, writing—original draft, writing—review and editing.

AC: Investigation, writing—original draft, writing—review and editing.

SK: Investigation, writing—review and editing.

LM: Conceptualization, validation, supervision, writing—original draft, writing—review and editing.

VT: Conceptualization, validation, supervision, writing—original draft, writing—review and editing.

AJG: Conceptualization, methodology, supervision, writing—review and editing.

The content and views expressed in this article are those of the authors and do not necessarily reflect those of the Government of Canada.
